# Subrepellent doses of Slit1 promote Netrin-1 chemotactic responses in subsets of axons

**DOI:** 10.1186/s13064-015-0036-8

**Published:** 2015-03-20

**Authors:** Isabelle Dupin, Ludmilla Lokmane, Maxime Dahan, Sonia Garel, Vincent Studer

**Affiliations:** University Bordeaux, IINS, UMR 5297, F-33000 Bordeaux, France; CNRS, IINS, UMR 5297, F-33000 Bordeaux, France; ENS, IBENS, INSERM U1024, CNRS UMR8197, 46 rue d’Ulm, 75005 Paris, France; Institut Curie, Laboratoire de Physico-Chimie, CNRS UMR 168, Université Pierre et Marie Curie-Paris 6, 75005 Paris, France; Centre de Recherche Cardio-thoracique de Bordeaux, U1045, CIC 0005, INSERM, University Bordeaux, F-33000 Bordeaux, France

**Keywords:** Axon guidance, Microfluidic, Netrin-1, Slit1, Thalamic neurons, Hippocampal neurons

## Abstract

**Background:**

Axon pathfinding is controlled by guidance cues that elicit specific attractive or repulsive responses in growth cones. It has now become clear that some cues such as Netrin-1 can trigger either attraction or repulsion in a context-dependent manner. In particular, it was recently found that the repellent Slit1 enables the attractive response of rostral thalamic axons to Netrin-1. This finding raised the intriguing possibility that Netrin-1 and Slit1, two essential guidance cues, may act more generally in an unexpected combinatorial manner to orient specific axonal populations. To address this major issue, we have used an innovative microfluidic device compatible not only with dissociated neuronal cultures but also with explant cultures to systematically and quantitatively characterize the combinatorial activity of Slit1 and Netrin-1 on rostral thalamic axons as well as on hippocampal neurons.

**Results:**

We found that on rostral thalamic axons, only a subthreshold concentration of the repellent Slit1 triggered an attractive response to a gradient of Netrin-1. On hippocampal neurons, we similarly found that Slit1 alone is repulsive and a subthreshold concentration of Slit1 triggered a potent attractive or repulsive behavioral response to a gradient of Netrin-1, depending on the nature of the substrate.

**Conclusions:**

Our study reveals that at subthreshold repulsive levels, Slit1 acts as a potent promoter of both Netrin-1 attractive and repulsive activities on distinct neuronal cell types, thereby opening novel perspectives on the role of combinations of cues in brain wiring.

**Electronic supplementary material:**

The online version of this article (doi:10.1186/s13064-015-0036-8) contains supplementary material, which is available to authorized users.

## Background

The assembly of neural circuits, a process essential to brain functioning, relies on the formation of precise axonal connections between neurons located in distinct regions. Axons can navigate across long distances and follow a complex trajectory to reach their appropriate target. Growing axons are guided along their journey by their environment via a motile structure located at their tip called the growth cone. Indeed, growth cone navigation and axonal extension require factors belonging to the superfamily of guidance cues, which promote the growth, attraction, or repulsion of axons in the nervous system. Distinct populations of neurons express specific combinations of receptors for guidance cues that enable them to respond specifically to sets of cues. While the effect of these cues has been individually tested, it is still largely unknown how growth cones integrate the information from the multiple cues they encounter to select their trajectory *in vivo*. To tackle this major issue, it is essential to develop novel methodological approaches that allow an integrative and quantitative analysis of chemotactic response of individual growth cone to combinations of molecular cues. Such an approach will provide key information to unravel the complex orchestration of neuronal connections by a limited set of guidance cues.

The guidance factor Netrin-1 constitutes a bifunctional cue that attracts some neurons and repels others [1]. This differential attractive or repulsive activity has been mainly explained by the fact that cells express different combinations of Netrin-1 receptors. However, it has also been proposed that adding another diffusible factor to a source of Netrin-1 could trigger a distinct response of the cell [2,3]. For instance, the repellent Slit is able to shut down the attractive effect of Netrin-1 during spinal cord midline crossing and motor neuron outgrowth [4,5]. Slit and Netrin can act synergistically to promote medial longitudinal fasciculus axon outgrowth [6]. It has also been described that Netrin-1 attractive activity on rostral but not intermediate thalamic axons is elicited by the addition of the repellent Slit1 [7].

However, which relative concentrations of Netrin-1/Slit1 trigger this combinatorial activity on rostral thalamic axons and whether it is specific to this axonal population remain to be determined. The lack of quantitative description is largely due to the difficulty in performing a precise analysis in conventional *in vitro* assays [8]. To solve this issue, we took advantage of an innovative microfluidic device enabling precise and parallel stimulation and observation of multiple neurons [9,10]. Importantly, the microchannels are separated from the culture chambers by a semi-permeable membrane. As result, there is no direct exposure of neurons to flows and the associated shear stress that are known to negatively affect neuronal development and survival [11]. Unlike the micropipette-based turning assay, the microfluidic device offers the possibility to probe the response of multiple growth cones. Furthermore, and in contrast to the Dunn chamber, it is compatible with culture of explants, thereby enabling the analyses of axons that grow poorly in dissociated cultures like thalamic axons.

Here, we show that the microfluidic device provides a unique system to quantitatively investigate combinatorial effects in growth cone turning. In rostral thalamic explants, Slit1 efficiently promoted Netrin-1 attraction at subthreshold repulsive doses, revealing a complex integration of Slit1 and Netrin-1 guidance activities. We furthermore found related combinatorial effects in hippocampal neurons: in addition to the specific repulsive function of Slit1 at high concentration, subthreshold level of Slit1 promoted either Netrin-1 attraction or repulsion depending on the nature of the substrate. Our study thus reveals that Slit1 promotes Netrin-1 chemotactic activity at subthreshold doses in several but specific populations of neurons, thereby opening novel perspectives on the integration of these two main guidance signals.

## Results

### An *in vitro* microfluidic guidance assay for axons

**Figure 1:**
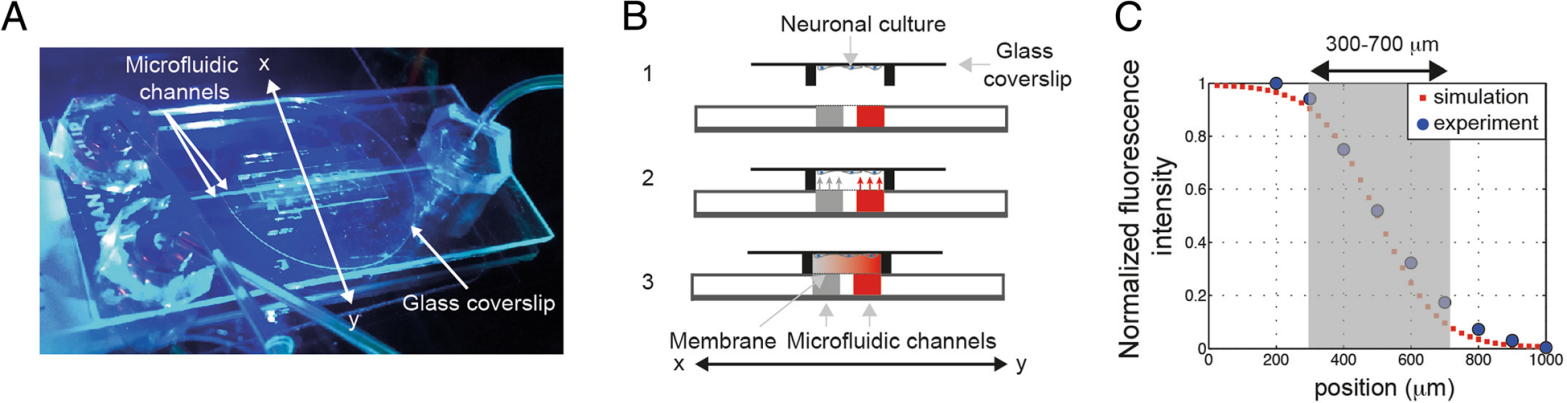
**Principle of the microfluidic device.** Top **(A)** and side **(B)** view of the microfluidic device. The explant is cultured in a microwell, and the neuronal culture coverslip is placed in contact with microchannels interfaced with a porous membrane for the experiment. A gradient forms over the microwell. **(C)** The concentration profile at the coverslip surface, perpendicular to the channel axis, in the center of a microwell, measured by TIRFM (blue dots) and obtained by simulation using a diffusion model (red squares).

**Figure 2:**
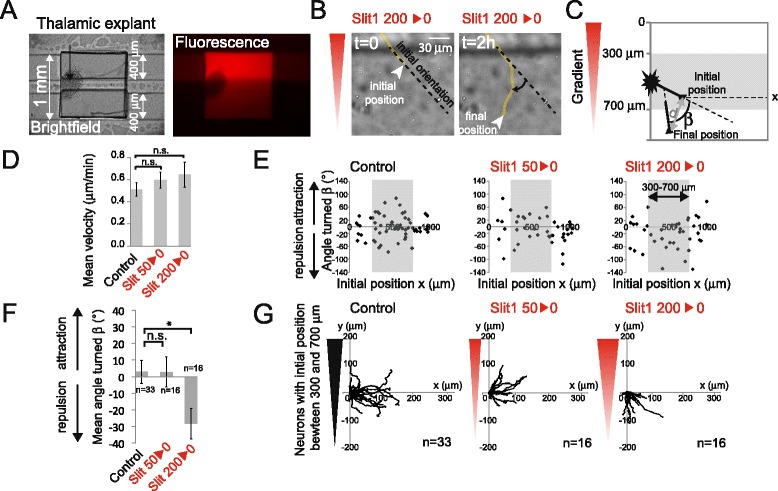
**Slit1 is repulsive for rostral thalamic axons at high concentration. (A)** Brightfield image (left) of a typical experiment showing the explant cultured in a microwell on top of the microfluidic channels and the corresponding fluorescence image (right). **(B)** Example of a growing axon turning down a Slit1 gradient. **(C)** Defining the angle turned (*β*), the distance between initial/final position (*d*), and the initial position (*x*). The angle turned was defined as positive for turns towards the gradient and negative for turns away from the gradient. **(D)** The mean velocity (± standard deviation) defined as the distance ‘*d*’ divided by the time. n.s.: *P* > 0.05, Mann–Whitney test in which each condition is compared to the control. **(E)** Scatter plot of the angle turned *versus* the initial position (*x*). **(F)** The mean angle turned (*β*) (±SEM) for axons in the different conditions, for initial positions between 300 and 700 μm. Statistical differences are indicated *P < 0.05, Kruskal Wallis test with Dunn’s correction. **(G)** Trajectory plots of growth cones in the different conditions, for initial positions between 300 and 700 μm.

To investigate the combinatorial effects triggered by guidance cues, we used a microfluidic device similar to those described in [9] made of two fluidic channels separated by a gap of 200 μm (Figures 1A,B,C and 2A). One channel (the reservoir) is filled with a solution of guidance factors while the other contains only buffer. As a result, a gradient is generated in the cell chamber by diffusion through the porous track-etched membrane (Figure 1A,B,C). Using a tetramethylrhodamine-labeled 70 kDa dextran, we characterized by total internal reflection fluorescence microscopy (TIRFM) the concentration profile at the location of the neuronal culture. The gradient shape, which is dictated by the geometry of the system, is in good agreement with the diffusion-based model described in [10] (Figure 1C) and Additional file 1: Figure S1 and Additional file 2: Figure S2. In the central part of the chamber (300 to 700 μm area, Figure 1C), the concentration profile is approximately linear with a relative slope of 2%, defined as the change in concentration along the width of the growth cone (10 μm) divided by the concentration *c*_*R*_ of factors in the reservoir. In our experiments, we only analyzed neurons located in this linear gradient area, and thus changing the concentration in the reservoir allowed us to simply change the gradient, characterized by the value of *c*_*R*_ (in ng/mL). We also wish to stress that numerical simulations and experiments (Additional file 1: Figure S1 and Additional file 2: Figure S2) show that the concentration profile is not significantly perturbed by the presence of neurons or explants in the chamber.

### Validation of the microfluidic guidance assay for thalamic neurons

To examine whether thalamic neurons could respond to gradients generated by this microfluidic device, rostral thalamic explants were cultured into PLL-laminin-coated microwells for 48 h and placed in contact with the microfluidic device at the onset of the experiment (Figure 2A). First, we applied gradients of Slit1 which is a well-known repellent for rostral thalamic neurons [7]. The position of the growth cone was followed every minute over a 2-h time period, and we assessed the mean velocity by measuring the net extension (Figure 2B,C, ‘d’) divided by the tracking period. We applied Slit1 gradients with different absolute slopes by changing the concentration of Slit1 in the reservoir (50 and 200 ng/mL). In all cases, the extension velocity of neurons was unchanged, suggesting that Slit1 does not affect growth rates of elongating neurons (Figure 2D). To quantify growth cone turning, we measured the turning angle (*β*), defined as the angle between the original and the final direction of the neuronal process (Figure 2C) [12]. This angle was positive for turns towards the gradient. In the control and 50 ng/ml Slit1 gradients, the distribution of angles appears random with a mean angle close to 0°, indicating an absence of chemotactic effect (Figure 2E,F,G). In 200 ng/ml Slit1 gradient, the distribution of angles was significantly shifted towards negative values, consistent with a chemorepulsion induced by high doses of Slit1 as also shown by the trajectories (Figure 2B,F,G). Finally, in our experiments, the initial orientation of the growing axons relative to the applied chemical gradient was not a significant parameter of their chemotactic response. Altogether, our results show that our microfluidic assay can be used to assess *in vitro* thalamic guidance and that a steep Slit1 gradient (200 ng/ml), but not a shallower gradient (50 ng/ml), is repulsive for rostral thalamic neurons.

### Low concentrations of Slit1 potentiate Netrin-1 attraction

**Figure 3:**
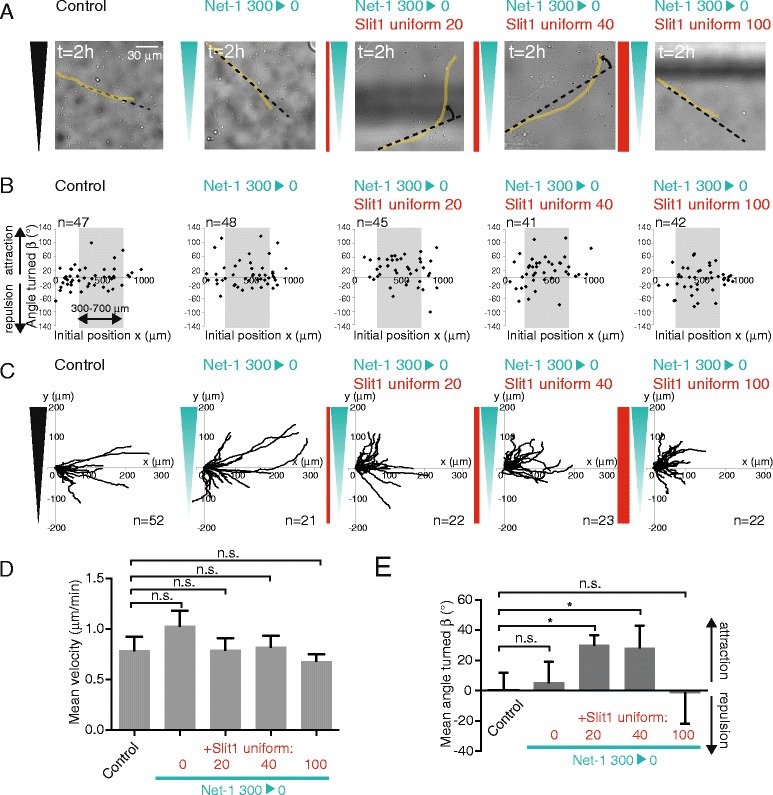
**Slit1 enables Netrin-1 attraction at low concentration. (A)** Last brightfield images of typical growing axons. The growing neurite (yellow line), the initial orientation (dark dotted line), and the angle turned (rotating arrow) are shown. Bar, 30 μm. **(B)** Scatter plot of the angle turned *versus* the initial position (*x*). **(C)** Trajectory plots of growth cones in the different conditions, for initial positions between 300 and 700 μm. **(D)** The mean velocity (±SEM). n.s.: *P* > 0.05, Mann–Whitney test in which each condition is compared to the control. **(E)** The mean angle turned (*β*) (±SEM) for axons in the different conditions, for initial positions between 300 and 700 μm. Statistical differences are indicated *P < 0.05, Kruskal Wallis test with Dunn’s correction.

As it has been previously shown that moderate levels of Slit1 enables Netrin-1 attraction for rostral thalamic neurons [7], we characterized *in vitro* neuronal responses to a gradient of Netrin-1 with increasing uniform doses of Slit1. In the absence of Slit1, a steep 300 ng/ml Netrin-1 gradient did not induce significant attraction on rostral thalamic neurons (mean turning angle = 9° ± 8°, Figure 3), consistent with previous results obtained by co-culture experiments [7]. Combining the Netrin-1 gradient with a uniform concentration of Slit1 triggered significant attraction for rostral thalamic neurons, from 20 ng/ml concentration of Slit1 (mean turning angle = 29° ± 8°, Figure 3) and up to 40 ng/ml (mean turning angle = 27° ± 9°, Figure 3), indicating that a low level of Slit1 is sufficient to induce the response of rostral thalamic neurons to Netrin-1. Further increase in Slit1 concentration (100 ng/ml) did not elicit Netrin-1-induced attraction (mean turning angle = −6° ± 8°) (Figure 3F). In all the conditions tested, the mean velocity remained unchanged, showing that the effect induced by the combination of Netrin-1 and Slit1 is not a growth effect (Figure 3D). Our data indicates that moderate levels of Slit1 (from 20 to 40 ng/ml) are sufficient and necessary to induce Netrin-1 attraction. In contrast, no chemotactic effect is observed when Slit1 is applied as a gradient in the same range of concentrations (5 to 45 ng/ml for the 50 ng/ml Slit1 gradient, see Figure 2F). Combining Netrin-1 gradient with a uniform 100 ng/ml Slit1 concentration inhibits the response to the Netrin-1, suggesting that Slit1 enables Netrin-1 attraction only in a range of low concentration.

### Slit1 potentiates Netrin-1 attraction of hippocampal neurons on PLL-laminin-coated substrate

**Figure 4:**
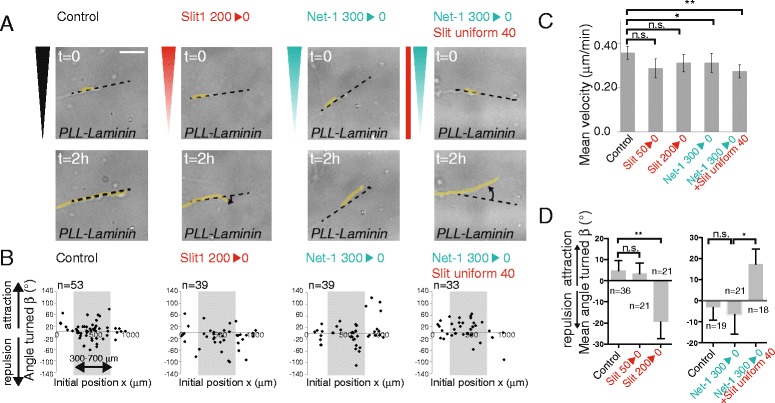
**Slit1 triggers Netrin-1 attraction for hippocampal neurons on PLL-laminin-coated substrates. (A)** Last brighfield images of typical growing axons on PLL-laminin-coated microwells. The growing neurite (yellow line), the initial orientation (dark dotted line), and the angle turned (rotating arrow) are shown. Bar, 20 μm. **(B)** Scatter plot of the angle turned *versus* the initial position (*x*). **(C)** The mean velocity (±SEM). *P < 0.05, **P < 0.01, Mann–Whitney test in which each condition is compared to the control. **(D)** The mean angle turned (*β*) (±SEM) for axons in the different conditions, for initial positions between 300 and 700 μm. Statistical differences are indicated *P < 0.05, **P < 0.01, Kruskal Wallis test with Dunn’s correction.

To directly test if Slit1 could also modulate Netrin-1 activity in other neuronal subtypes, we applied gradients of guidance factors on dissociated hippocampal neurons (Figure 4A). To compare the response of hippocampal neurons to Slit1 and Netrin-1 with those obtained with thalamic neurons, we first performed culture on a PLL-laminin-coated substrate (although hippocampal neurons are classically cultured on PLL-coated dishes). Axon velocities remained unchanged by the addition of guidance cues, except for the 300 ng/ml Netrin-1 gradient and for the combination of a 300 ng/ml Netrin-1 gradient and a uniform concentration of 40 ng/ml Slit1 (Figure 4C). A significant repulsion can be observed for hippocampal neurons submitted to a 200 ng/ml Slit1 gradient (mean turning angle = −19° ± 8°) (Figure 4A,B,D), whereas a gradient of 300 ng/ml Netrin-1 did not induce significant chemotactic activity (mean turning angle = −7° ± 9°) (Figure 4A,B,D). Similarly to what we observed in rostral thalamic neurons, we found that hippocampal neurons growing on PLL-laminin-coated microwells were significantly attracted by the combination of a 300 ng/ml Netrin-1 gradient and a uniform concentration of 40 ng/ml Slit1 (mean turning angle = 17° ± 7°, Figure 4A,B,D). This shows that the effect triggered by the combination of Netrin-1 and Slit1 is not specific to rostral thalamic neurons and also exists in hippocampal neurons.

### The effect of laminin switches the response of hippocampal axons to Netrin-1

**Figure 5:**
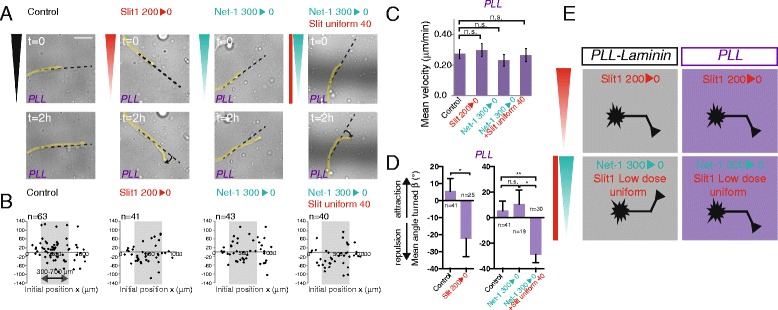
**Slit1 triggers Netrin-1 repulsion for hippocampal neurons on PLL-coated substrate. (A)** Last brighfield images of typical growing axons on PLL-coated microwells. The growing neurite (yellow line), the initial orientation (dark dotted line), and the angle turned (rotating arrow) are shown. Bar, 20 μm. **(B)** Scatter plot of the angle turned *versus* the initial position (*x*). **(C)** The mean velocity (±SEM). n.s.: *P* > 0.05, Mann–Whitney test in which each condition is compared to the control. **(D)** The mean angle turned (*β*) (±SEM) for axons in the different conditions, for initial positions between 300 and 700 μm. Statistical differences are indicated *P < 0.05, Kruskal Wallis test with Dunn’s correction. **(E)** Schema showing the responses of hippocampal neurons to different stimulations depending on the nature of substrate coating. On PLL and PLL-laminin-coated microwells, hippocampal neurons are repelled by a Slit1 gradient at high concentration. When submitted to the combination of a Netrin-1 gradient with a low uniform concentration of Slit1, the response of hippocampal growth cones is shifted from repulsion on PLL-coated substrate to an attraction on PLL-laminin-coated substrates.

We performed in parallel the same experiments with hippocampal neurons growing on a PLL-coated substrate, which is the classical culture condition for these neurons. Similarly, on PLL or on a PLL-laminin substrate, a repulsion can be observed for hippocampal neurons submitted to a 200 ng/ml Slit1 gradient (mean turning angle = −22° ± 11° on PLL substrate) while a 300 ng/ml Netrin-1 gradient did not trigger significant chemotactic activity (mean turning angle = 10° ± 12° on PLL substrate) (Figure 5A,B,D). Strikingly, the response to the combination of both cues was shifted from attraction to repulsion when hippocampal neurons were growing on PLL substrate (mean turning angle = −22° ± 11°, Figure 5A,B,D) suggesting that the extracellular matrix can regulate the response of growth cones to diffusible cues. This shift was specific to the combination Netrin-1/Slit1 since Slit1 alone elicited a repulsive response in hippocampal axons on either PLL or PLL-laminin-coated substrate. In all the conditions, the axons grew at similar velocities (Figures 4C and 5C). Altogether, our results show that Slit1 enables the chemotactic activity of Netrin-1 in hippocampal neurons, irrespective of whether this activity is attractive or repulsive (Figure 5E).

## Discussion

Characterizing the combinatorial activity of guidance cues [7] is essential to grasp the logic governing the assembly of neural circuits *in vivo*. However, it has remained a real challenge to use quantitative *in vitro* assays that are compatible with cultured dissociated neurons as well as with explants. In particular, neurons are highly sensitive to flow in their environment, and explants often provide controlled growth conditions that are close to physiological contexts. Here, we have taken advantage of a shear-free microfluidic device in which the culture system is decoupled from the microfluidic circuit and thereby overcomes the usual difficulties of culturing neurons in closed microenvironments [9,10]. With this microfluidic assay, the response of groups of neurons to well-controlled profiles of one or more chemical cues can be monitored at the single cell level. As the quantitative chemotaxis assay to assess axon guidance in three-dimensional gels [13], this microfluidic device thus constitutes a major technological advance to the study of the integrative mechanisms underlying the axonal response to individual or combination of extracellular signals.

**Figure 6:**
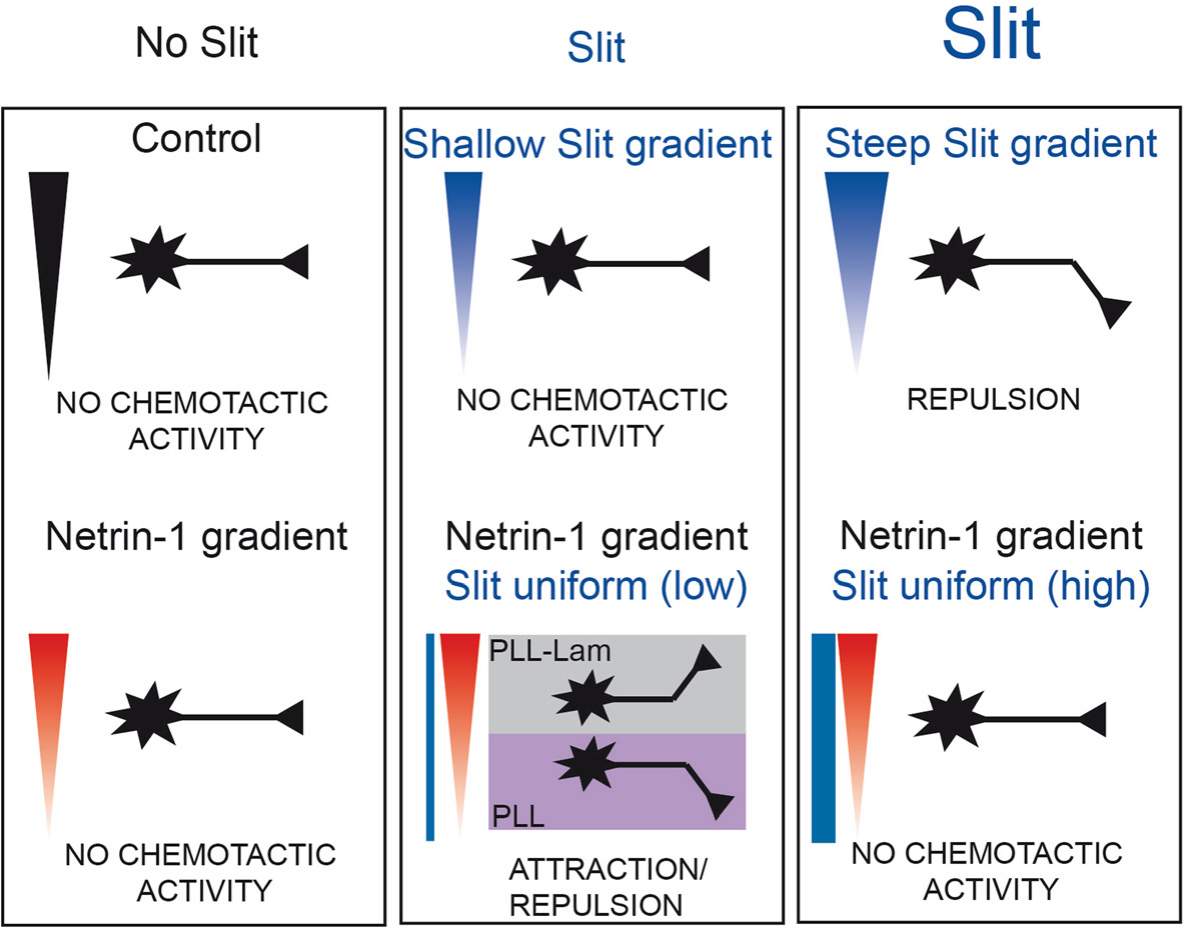
**Subthreshold levels of the repellent Slit1 elicited Netrin-1 attraction or repulsion in specific axonal populations.**

Using these shear-free microfluidic devices, we investigated in detail the unexpected combinatorial activities of Slit1 and Netrin-1 on rostral thalamic axons [7]. We found that a steep Slit1 gradient (200 ng/ml in the reservoir, concentration range: 20 to 180 ng/ml) but not a shallower Slit1 gradient (50 ng/ml in the reservoir, concentration range: 5 to 45 ng/ml) promoted repulsion (Figure 6). In combinatorial conditions, we observed that Slit1 only elicited Netrin-1 attraction at subthreshold repulsion levels (Figure 6). Thus, in the absence of other guidance cues that may have a stronger effect on thalamic axons, Slit1 dictates the neuronal response to a Netrin-1 gradient. This observation is particularly relevant to assess axon pathfinding during brain development since growth cones can become responsive to Netrin-1 gradient only upon reaching a region of moderate Slit1 concentration.

To estimate the general relevance of this finding, we examined hippocampal neurons that have been previously shown to respond to both guidance cues. Interestingly, we found that not only do hippocampal neurons show a specific response to the combination of Netrin-1 and Slit1 but that Slit1 promotes the chemotactic activity of Netrin-1, either attractive or repulsive. This observation indicates that the combinatorial effect is not specific to rostral thalamic neurons and is thus unlikely to constitute a general neuronal property. Indeed, it had been previously shown that several neuronal populations respond to Netrin-1 and that intermediate thalamic axons are not attracted by the combination of Netrin-1 and Slit1 [7]. We thus suspect that the promoting effect of Slit1 on Netrin-1 activity may be specific to some neuronal populations, thereby expanding the repertoire of possible responses to identical signals. A recent study shows that the attractive response of rostral thalamic axons to the combination of Netrin-1 and Slit1 is determined by the expression and function of Robo1 and FLRT3, a novel coreceptor for Slit1 [14]. This work provides an interesting mechanism to explain the cell-type specificity of the combinatorial activity. Consistently, FLRT3 is expressed at high levels in hippocampal neurons [14]. Slit is also able to bind Netrin-1 [15], and the association of the two molecules and possibly the regulation of the interaction could be implicated in the combinatorial effect of Netrin-1 and Slit1.

In hippocampal neurons, we found a striking combinatorial effect that varied depending on the substrate on which the neurons were cultured. Nevertheless, we observed a limited effect of Netrin-1 on hippocampal neurons cultured either on PLL or PLL-laminin. In contrast to our findings, [16] reported that hippocampal neurons growing on a PLL-coated substrate are attracted by a Netrin-1 gradient using the micropipette-based assay whereas we did not find any chemotactic activity of Netrin-1 in the same conditions. Yet, a recent study analyzed the response of hippocampal neurons depending on the levels of the Netrin-1 receptor UNC5A [17]. Growth cones that are attracted or repelled by Netrin-1 are respectively UNC5A-negative and UNC5A-positive, each subpopulation representing half of the growth cones. These findings are in agreement with our results, showing no significant chemotactic activity of a Netrin-1 gradient on a heterogenous hippocampal population. We have also shown that changing the nature of the extracellular matrix changes the polarity of the turning response of hippocampal neurons to the combination of Netrin-1 and Slit1. These findings are reminiscent of data obtained in Xenopus neurons in which laminin-1 converts netrin-mediated attraction into repulsion [3]. While the switching effect is inverted compared to our observations, probably due to the different neuronal types used, it confirms the key role of the extracellular matrix in regulating axon guidance. Although future experiments are needed to precisely understand the mechanisms, our study demonstrates that Slit1 promotes the chemotactic activity of Netrin-1, either attractive or repulsive. Slit1 acts at subthreshold repulsive levels, thereby revealing complex integrated responses to a combined source of Slit1 and Netrin-1. These experiments set the foundation for large-scale assessments of combinatorial activities of extracellular cues and provide a quantitative framework to unravel the complex wiring of neuronal networks.

## Conclusions

Using a shear-free microfluidic assay, we quantitatively describe the combinatorial effect of Netrin-1 and Slit1 on axon guidance. Our results show that Slit1 elicited Netrin-1 attraction at subthreshold levels in rostral thalamic neurons. This combinatorial effect was also observed in hippocampal neurons, and we showed that the presence or absence of laminin on the growing substrate switches hippocampal response from attraction to repulsion. Altogether, our study reveals that for specific axonal populations, the combination of Slit1 and Netrin-1 elicits complex axonal responses depending on their relative concentrations, thereby opening new perspectives for characterizing the roles of axon guidance factors *in vivo.* In addition, we believe that our results demonstrate the advantages of this microfluidic assay to investigate axon guidance and to decipher the complex rules that govern the axonal response to graded signals. This *in vitro* assay has clear potential application to address chemotactic effects triggered by single or combined extracellular cues.

## Methods

### Membrane-based device microfabrication

The devices were fabricated as described in [9] with some modifications for membrane assembly and the connectors. After obtaining a stiff micropatterned layer of NOA 81 (Norland Products Inc., Cranbury, NJ, USA), with channel network, a glass side was pressed under the microcircuit and a 3 × 5 mm^2^ membrane (polyester hydrophilic track-etched membrane, 12 mm thickness, 400 nm hole diameter, it4ip, Louvain-la-Neuve, Belgium) was aligned and pressed on top of the NOA 81 layer. An additional UV illumination (2 min, 24 mW/cm2) was applied for irreversibly bonding the assembly. Before being used for culture experiment, the devices were soaked in water for at least 2 days.

### Cell culture wells fabrication

PDMS solution was spin-coated onto a silicon wafer at 1,600 rpm for 60s. The PDMS thin film was partially cured for 30 min at 70°C. 1 mm × 1 mm culture microwells were cut in a gel film (PF Gel-Film, gel thickness 150 μm, Gel-Pack, Hayward, CA, USA) with a vinyl plotter (Graphtec Robo CE-4000, Graphtec America Inc., Irvine, CA, USA). The square microwells were then pressed on top of the PDMS film. An additional curing phase of at least 2 h at 70°C was applied to irreversibly bond the assembly of the two levels of PDMS and gel film. The chambers were sterilized with deep UV treatment (185 and 254 nm, 13 mW/cm2, 5 min). The chambers were then washed two times with ethanol, one time with sterilized deionized water. Cell culture chambers were coated with 100 μg/ml PLL (Sigma-Aldrich, St. Louis, MO, USA), rinsed three times with sterilized deionized water and air-dried during at least 2 h. Cells were either added at this step (PLL-coated coverslips) or after an additional overnight coating with 10 μg/ml laminin (Sigma-Aldrich, St. Louis, MO, USA) at 37°C.

### Gradient quantification

Tetramethylrhodamine 70 kDa neutral dextran (Invitrogen, Carlsbad, CA, USA) was injected at 50 μg/ml in one of the microfluidic channel. Images of the gradient in the microwell at the close vicinity of the coverslip were acquired by TIRFM on an inverted microscope (Eclipse Ti, Nikon Corporation, Tokyo, Japan) equipped with an oil-immersion objective (100×, TIRF 1.49 NA) and a laser illumination arm (Nikon TIRF, Nikon Corporation, Tokyo, Japan) and a 561 nm laser (Vortran Laser Technology Inc., Sacramento, CA, USA). The red fluorescence of the sample was recorded on an EMCCD camera (Photometrics Evolve 512, Photometrics, Tucson, AZ, USA). A discrete map of the local fluorophore concentration at the surface of the microwell could thus be obtained. The plot on Figure 1C corresponds to a section of this map in the middle of the microwell and is in good agreement with the simulated concentration profile [10].

### Mouse thalamic neurons explants

Coronal slices of different levels of OF1 (Oncins France 1) mouse at embryonic day E13.5 (Charles River Laboratories, Bois des Oncins, France) were prepared as previously described [18]. Rostral thalamic explants were dissected from these rostral telencephalic slices and cultured into PLL-laminin-coated microwells in neurobasal medium supplemented with 1× NeuroMix (PAA, Pasching, Austria), 20 mM glucose (Sigma-Aldrich, St. Louis, MO, USA), 2 mM glutamine, and 1× pen-strep (both from Invitrogen, Carlsbad, CA, USA) for 48 h.

### Rat hippocampal neurons culture

Hippocampi were dissected from Sprague–Dawley rat (Janvier, Saint Berthevin Cedex, France) at embryonic day E18 and dissociated using trypsine and mechanical trituration. Hippocampal cells were plated at a density of 130 cells/mm^2^ on PLL or PLL-laminin-coated coverslips in neurobasal medium supplemented with 1× NeuroMix (PAA, Pasching, Austria), 20 mM glucose (Sigma-Aldrich, St. Louis, MO, USA), 2 mM glutamine, and 1× pen-strep (both from Invitrogen, Carlsbad, CA, USA).

### Microfluidic experiments and image acquisition

Gradients of recombinant Netrin-1 and Slit1 proteins (R&D Systems, Minneapolis, MN, USA) were produced by injecting medium (without pen-strep) with and without guidance factor in the two entries, respectively. Flows were controlled by a pressure regulator (Fluigent MFCS-8C, Fluigent, Paris, France). A uniform concentration of guidance factors was produced by injecting medium with the same concentration of guidance factor at the two entries. Guidance cues were diluted in culture medium supplemented with 20 mM HEPES (Invitrogen, Carlsbad, CA, USA). Cell culture coverslips were placed upside down onto the microfluidic device on a heated microscope stage and sealed using vacuum grease. Brightfield images of neurons were acquired every minute for 2 h on an upright macroscope (AZ100, Nikon Corporation, Tokyo, Japan) equipped with an objective (5× dry, N.A. 0.5; Nikon Corporation, Tokyo, Japan) and a charge-coupled device camera (CoolSNAP HQ2, Photometrics, Tucson, AZ, USA). Images were collected with the open-source Micro-Manager software (RRID:nlx_155812). We recorded the response of neurons growing in one culture microwell (1 mm × 1 mm) per experiment.

### Image analysis

Image analysis and measurements were performed with the open-source ImageJ software (RRID:nif-0000-30467). The initial position of the growth cone (*x*) in the culture chamber was measured. The turning angle (*β*) was defined as the angle between the original and the final direction of the neuronal process. Only neurites showing a net extension of at least 15 μm during the imaging period were included in the analysis. The distance *d* from the initial and final positions of the growth cone was measured. The tracking period is the time between the beginning and the end of the tracking for each analyzed growth cone. The mean velocity was defined as the distance *d* divided by the tracking period. Growth cones that collapsed during the time of the experiment were tracked only if they exhibited growth during at least 1 h. The position of growth cones was manually tracked using the Manual Tracking plugin. Eventual drifts in *xy* directions were corrected by calculating the position of the growth cone relative to a fixed spot. For each condition, at least three independent experiments were performed. Statistical differences were determined using either the Mann–Whitney test or the Kruskal-Wallis test with Dunn’s correction. Statistical significance is defined as *P* < 0.05.

### Ethical issues

The Animal Care and Use Committee (Bordeaux University, Bordeaux, France) approved our detailed experimental protocol including animal use procedures under the reference no. 50110017-A.

## Additional files

Additional file 1: Figure S1. A and B show the result of a numerical simulation of the normalized concentration profile of the guidance cue, obtained in the cross section of the neuronal culture chamber without (A) or with (B) an explant positioned at the coverslip. The presence of an explant only slightly perturbs the concentration gradient at the very close proximity of the explant, as can be seen on C), a plot of the normalized concentration profile with (in green) or without an explant. The green curve is interrupted because the concentration of the guidance cue is undefined at the position of the explant. Additional file 2: Figure S2. A) A hard cylinder mimicking a large explant was microfabricated in photoresist (SU8-2100 Microchem USA) (diameter approx. 100um, height approx. 100um) on a glass coverslip inside a microchamber by conventional photolithography. A microscopy picture of the chamber, the artificial explant, aligned on top of the microchannels is shown. B) The concentration profile at the coverslip was quantified using the same protocol than on Figure 1C. The profile is measured at 3 different positions along the microchamber. The positions of the measured profiles is shown on A. The theoretical profile is also represented. These results show that the experimental concentration profile is close to the theoretical one even in the presence of a large object inside the microchamber.

## Abbreviations

PDMS: Polydimethylsiloxane
PLL: Poly-L-lysine
SEM: Standard error of the mean
TIRFM: Total internal reflection fluorescence microscopy
