# Supplementary figures and images for: Subrepellent doses of Slit1 promote Netrin-1 chemotactic responses in subsets of axons
Source: Neural Dev. 2015 Mar 20;10:5. doi: 10.1186/s13064-015-0036-8 (PMC4373007; doi:10.1186/s13064-015-0036-8)

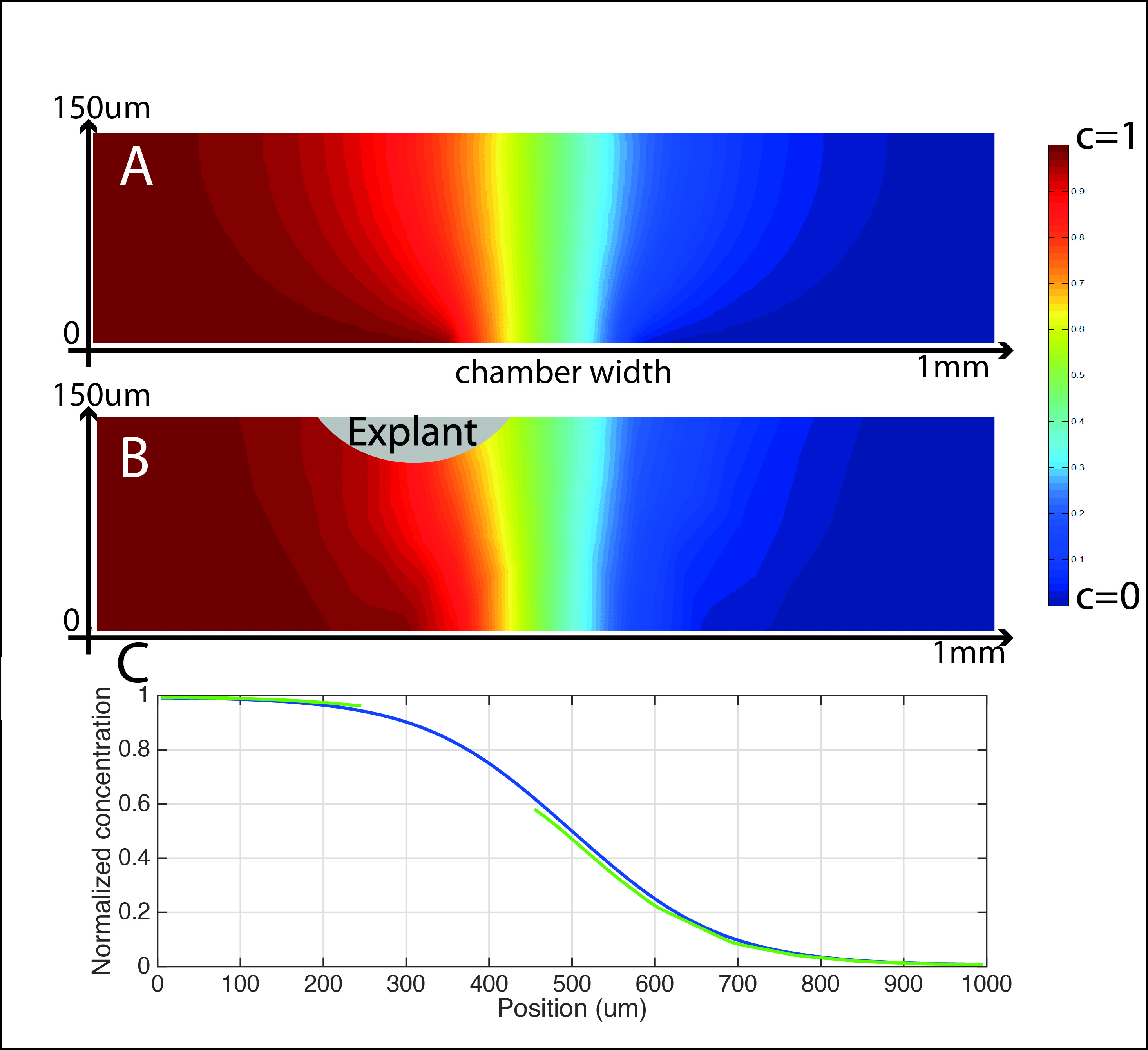

Supplement: Additional file 1: Figure S1. — A and B show the result of a numerical simulation of the normalized concentration profile of the guidance cue, obtained in the cross section of the neuronal culture chamber without (A) or with (B) an explant positioned at the coverslip. The presence of an explant only slightly perturbs the concentration gradient at the very close proximity of the explant, as can be seen on C), a plot of the normalized concentration profile with (in green) or without an explant. The green curve is interrupted because the concentration of the guidance cue is undefined at the position of the explant. [file 13064_2015_36_MOESM1_ESM.jpeg]
