# Supplementary material for: Subrepellent doses of Slit1 promote Netrin-1 chemotactic responses in subsets of axons
Source: Neural Dev. 2015 Mar 20;10:5. doi: 10.1186/s13064-015-0036-8 (PMC4373007; doi:10.1186/s13064-015-0036-8)
Supplement: Additional file 2: Figure S2. — A) A hard cylinder mimicking a large explant was microfabricated in photoresist (SU8-2100 Microchem USA) (diameter approx. 100um, height approx. 100um) on a glass coverslip inside a microchamber by conventional photolithography. A microscopy picture of the chamber, the artificial explant, aligned on top of the microchannels is shown. B) The concentration profile at the coverslip was quantified using the same protocol than on Figure 1C. The profile is measured at 3 different positions along the microchamber. The positions of the measured profiles is shown on A. The theoretical profile is also represented. These results show that the experimental concentration profile is close to the theoretical one even in the presence of a large object inside the microchamber. [file 13064_2015_36_MOESM2_ESM.pdf]

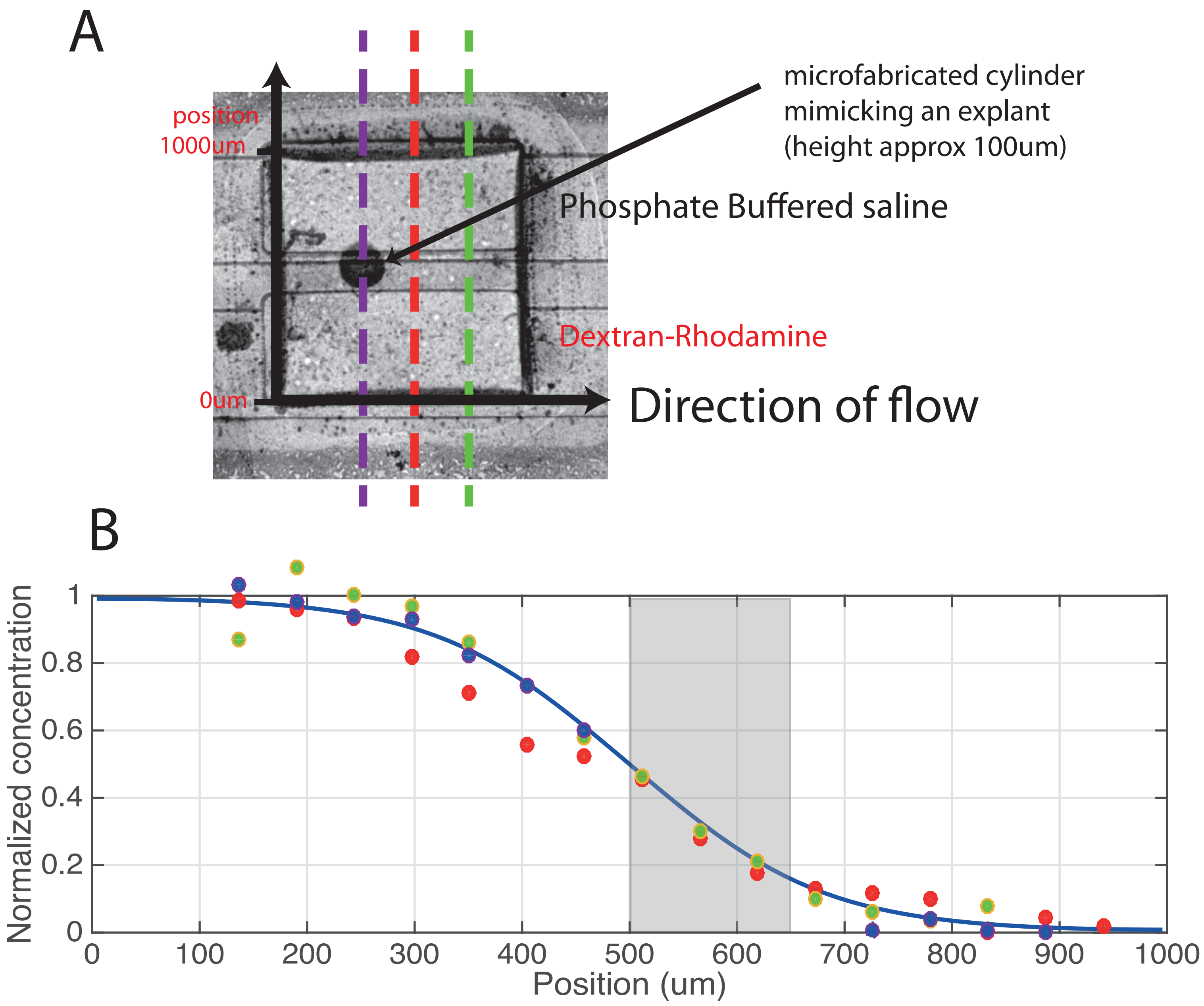
A
position
1000um
0um
microfabricated cylinder
mimicking an explant
(height approx 100um)
Phosphate Buffered saline
Dextran-Rhodamine
Direction of flow
B
Normalized concentration
0
0.2
0.4
0.6
0.8
1
0
100
200
300
400
500
600
700
800
900
1000
Position (um)
